# Experiences of family caregivers of children with cancer while receiving home-based pediatric palliative care in Indonesia: a qualitative study

**DOI:** 10.1186/s12904-022-00986-5

**Published:** 2022-06-07

**Authors:** Christantie Effendy, Deby Kristiani Uligraff, Selvia Harum Sari, Fany Angraini, Lynna Chandra

**Affiliations:** 1 Department of Medical Surgical Nursing, Faculty of Medicine, Public Health and Nursing, Universitas, Gadjah Mada, Yogyakarta, Indonesia; 2 Department of Nursing, Sekolah Tinggi Ilmu Kesehatan Tarumanagara, Jakarta, Indonesia; 3 Rachel House, Jakarta, Indonesia

**Keywords:** Experiences, Family caregivers, Children with cancer, Home-based pediatric palliative care, Indonesia

## Abstract

**Background:**

Caring for children living with life-threatening and life-limiting illnesses can be challenging. Parents’ roles as the main caregivers can be complex with extensive responsibilities. The experiences of family caregivers can provide key insights into the provision of home-based Pediatric Palliative Care (PPC) for seriously ill children. This study is aimed at exploring the experiences of family caregivers of children diagnosed with cancer while receiving home-based PPC.

**Methods:**

This was a qualitative study. This study used semi-structured interviews which were audio-recorded with family caregivers of twelve children diagnosed with cancer who had received home-based PPC. The interviews were transcribed verbatim. The data were analyzed using qualitative content analysis. The reporting of the study was based on the Consolidated Criteria for Reporting Qualitative Research (COREQ) checklist.

**Results:**

Three main themes emerged: (1) The implementation of home-based PPC; (2) The benefits of home-based PPC; and (3) The family caregivers’ hopes of the home-based PPC service and their impressions of home-based PPC. The implementation of home-based PPC described the experiences of family caregivers in receiving home-based PPC provided by nurses with particular attention to the bio-psychosocial-spiritual aspects. Family caregivers experienced several benefits from the home-based PCC service, where holistic care was provided for both the patient and the family. Family caregivers shared their hopes prior to receiving support from competent health care professionals to care for their sick child at home and improve the child's quality of life. They confirmed that these hopes were fulfilled through the home-based PPC service delivered by Rachel House.

**Conclusions:**

Home-based PPC provides several benefits with a positive impact for both the children diagnosed with cancer as well as their families. Nurses involved in the home-based PPC service provide holistic care with a family-centered approach. We believe that children with terminal illnesses and their families need and deserve home-based PPC during difficult times.

## Introduction

Caring for children living with life-threatening and life-limiting illnesses can be challenging [1]. Due to the prolonged and complicated disease trajectory, parents as the main caregivers experience extensive emotional, physical, psychological, technical and nursing demands [2]. Care can also become more challenging when a child is facing imminent death [3]. To support a family dealing with distressing circumstances while caring for a severely ill child, the palliative care (PC) model has been proposed as a holistic and sensitive approach to help reduce families’ and patients’ suffering [4, 5].

Home-based PC has been widely acknowledged as the holistic care model for patients living with life-threatening and life-limiting illnesses that could result in prolonged suffering [5]. The World Health Organization (WHO) guidelines emphasized that Pediatric Palliative Care (PPC) should begin at the time of diagnosis and be made available for every severely ill child and their family in both hospital and home settings [5]. Home-based PC aims to improve the quality of life of patients and their families by alleviating the devastating symptoms throughout the trajectory of the disease [6]. For children with life-threatening illnesses, PPC is essential [7, 8].

The development of PPC in Indonesia began in 2006 and the home-based service has been developing slowly since then [9]. Several hospitals are currently providing the service, albeit still mainly in Jakarta, the capital city of Indonesia. There is a non-profit organization, Rachel House, that provides home-based PPC for children under 18 years old living with serious illnesses such as cancer and HIV/AIDS [9]. The service is delivered by a group of PPC-trained nurses and receives patient referrals from major hospitals mostly around Jakarta. The nurses provide care and support not only for the children, but also for their families [9, 10].

Acceptance of PC in Indonesia remains a challenge due to cultural barriers. Many parents associate PC with care for the dying. Accordingly, accepting PC service may be interpreted as “giving up the fight” or going against the will of God, leaving them with feelings of guilt and regret, or even opening themselves up to criticism from family members. However, home-based PPC as a model of care is gaining acceptance since home is often the preferred place of care for parents with children living with life-threatening illness [11]. Recent research also showed that more children with life-limiting illness are receiving care at home [12].

Home-based PPC is preferred for children and their families because it is likely to improve the children’s quality of life and their health outcomes (e.g. pain, symptom management and anxiety) and minimize the hospital costs for extended care [13]. Furthermore, it was reported that home-based PPC may reduce caregivers’ psychological burden, helping families manage their sense of loss and grief, leading to a better quality of life [14, 15]. Home-based PPC is also essential to ensure a continuity of care and optimize quality of life throughout the disease trajectory, transitioning patients smoothly between hospital care to home [16].

Other benefits of home-based PPC are likely to emerge as we delve deeper into family caregivers’ experiences with the service, providing personal insights that can be considered as the key impressions of home-based PPC [17]. Research conducted by Gurková et al. [17] highlighted that each family’s experience in caring for a child dying from cancer is unique. The understanding of their experiences can be useful in developing a more effective strategy towards improving the quality of life of children living with a life-threatening illness and their families. This more personalized approach could also help improve the understanding of patient preferences, quality management in healthcare and public accountability [18]. This study is aimed at exploring the experiences of family caregivers of children living with cancer while receiving home-based PPC.

## Methods

This qualitative study was conducted with a phenomenological design [19]. This methodology is considered to be the most appropriate approach to explore human lived experiences. The data was collected through in-depth interviews allowing the researchers to gain insights into the caregivers’ experiences while receiving home-based PPC [2, 19]. The reporting of the study was based on the Consolidated Criteria for Reporting Qualitative Research (COREQ) checklist [20].

### Sample and setting

Purposive sampling technique was used to determine and recruit the family caregivers who were interviewed [21]. The sampling method was initiated by defining certain characteristics of targeted participants based on the inclusion criteria, and then applying the exclusion criteria (Table 1) [2, 19–21]. This method is considered to be the appropriate design to conduct a phenomenological study, which is aimed to explore participants’ experience. Based on the criteria set and considering the data saturation process, twelve family caregivers were selected to be interviewed regarding their experiences with home-based PPC service. The number of participants was decided when the data were saturated, where no further new information emerged in the code list. The decision was reached based on discussions among the research team. While data saturation was reached at the eleventh participant, one additional participant was added to ensure that the data was saturated.

**Table 1:** Participants Inclusion and Exclusion Criteria

**Inclusion criteria**	1.Family caregiver of a child living with end-stage cancer, no longer responding to curative treatment (as mentioned in the oncologist’s referral letter) 2.Patient is either a former patient or currently receiving HBPC from Rachel House 3.Patient has received a minimum of ten visits by Rachel House, either through home visits or telemedicine 4.Family caregiver agrees to be interviewed and to be involved in the research study 5.Written consent form was duly completed
Exclusion criteria	Family caregivers with difficulties in communication, for reasons of either dealing with ongoing emotions of grief and loss, or other emotional challenges such as denial, anger, and difficulties in moving on

### Ethical acknowledgement

This study was ethically approved by the Medical and Health Research Ethics Committee of the Faculty of Medicine, Public Health and Nursing of *Universitas* Gadjah Mada, Indonesia (number: KE/FK/0001/EC/2021) and the Health Research Ethics Committee Ngudia Husada Madura School of Health Sciences, Indonesia (number: 724/KEPK/STIKES-NHM/EC/XII/2020). All participants signed an informed consent form prior to data collection and anonymity was assured by assigning an identification number to each participant.

### Data collection

Data were collected using in-depth interviews from January to June 2020. The recruitment process used a personal approach toward participants who met the criteria [21]. Participants who expressed interest to participate were invited to be involved in the study. The interview was conducted for 40–90 min. At the beginning of each interview, the interviewers explained the aim of the study and data collection techniques. The informed consent was obtained before proceeding with the individual interviews. All the interviews were done by using a semi-structured interview guide (Table 2), audio-recorded, and transcribed verbatim. All interviews were conducted in Bahasa Indonesia (the national language). The interview guidelines were developed by panel discussions [22] among the researchers, including the principal investigator with expertise in qualitative research methods and palliative care, and three nurses at Rachel House who are active in providing home-based PPC [22].

**Table 2:** Open-Ended Questions

Guide questions	Probes
-What were your thoughts about your child’s condition? -What were the most challenging things in the care of your child living with cancer?	Why did you feel that way? Could you tell me more about that?
-What were your thoughts about taking care of your child with complex symptoms at home? -What were your feelings and thoughts when the doctor referred your child to home-based palliative care?	Could you tell me more about that? What worried you the most?
-What support did you expect to get from the home-based palliative care team? -What problems were troubling you the most when taking care of your child living with cancer?	Could you explain more? What were the challenges that you frequently encountered and emotions that you felt while receiving the assistance of home-based palliative care? For example, physical symptoms of your child (fever, pain), emotional situations (sadness), social challenges (feeling lonely), economic challenges (hospital expenses), spiritual issues (angry to God)
-(Depending on the answers from the previous questions): How did Rachel house help you overcome these problems? -What new information or skills did you get after receiving home-based palliative care from Rachel House’s nurses? -How was your child’s condition while receiving the home-based PPC provided by Rachel House?	What kind of assistance was most helpful in solving these problems? Could you explain more? How did you learn or able to apply the lessons? Did the home-based PPC meet your expectations?
-How did the home-based PPC help you in your decision-making process?	-If there were conflicts among family members prior to receiving care, was Rachel House’s nurse able to help you in any way? -How did Rachel House’s nurse help you to make decisions regarding your child’s care?
For family caregivers whose child has passed away: -How did Rachel House’s nurse help you in the preparation towards death and dying process? -How did Rachel House’s nurse support you during your bereavement process after losing your child?	Could you please elaborate

### Data Analysis

The data of this study were analyzed with qualitative content analysis, which is characterized by de-contextualization and re-contextualization and generally comprises the following steps: selecting the unit of analysis, coding all the data, revising the coding rules (if necessary), creating and defining the categories, revising the category scheme, and constructing themes [23–26]. First, data were transcribed after each interview was completed, followed by familiarization of data through re-reading of the transcripts by the research team members (DK, SH, and NJ). Second, at least two research team members met to generate the initial coding of the data. This process was completed before interviewing the next participant. Third, the team (DK, SH, NJ, FA and CE) met for recoding and clustering of the codes into potential categories. Fourth, the categories were analyzed to construct themes by the team. All transcripts were in Bahasa Indonesia, which at the reporting stage was translated into English by the team and proofread by a native English speaker.

### Trustworthiness

During the study process, the researchers took several strategies to maintain the rigor of this study [22, 25]. Member checking and investigator triangulation were used to ascertain the credibility of the findings. Agreement prior to the data analysis was achieved through peer debriefing and regular meetings among the research members and consultations with the principal investigator. The confirmability of this study was ensured by employing reflexivity for transparency and consistency throughout the analysis process. Furthermore, journaling and extensive meetings within the research team were conducted regularly for communicating and discussing the various perspectives towards data interpretation. The researchers employed an audit trail to ensure the dependability of this study by field noting and journaling throughout the process. The researchers used thick description and purposive sampling technique to establish the transferability of findings.

## Results

There were twelve participants involved in this research. All of the participants were female (mothers) except for one male (father). The age ranged from 20 to 50 years old. Seven of the participants were not working and the other five were working. The participants were parents whose children were living with cancer, acute lymphocytic leukemia (ALL) (*n* = 3; 25%), retinoblastoma (*n* = 2; 16.67%), neuroblastoma (*n* = 2; 16.67%), medulloblastoma (*n* = 2; 16.67%), rhabdomyosarcoma (*n* = 2; 16.67%), and atypical meningioma (*n* = 1; 8.33%). All children had died by the time the interviews were conducted (Table 3).

**Table 3:** Characteristic participants (*n* = 12)

Characteristics	*n*	%
Family caregiver		
Mother	11	91.67
Father	1	8.33
Education level		
Primary school	1	8.33
High School	7	58.33
Diploma and Graduate	2	16.67
Post Graduate	2	16.67
Children Diagnosis		
Acute lymphocytic leukemia (ALL)	3	25
Retinoblastoma	2	16.67
Neuroblastoma	2	16.67
Medulloblastoma	2	16.67
Rhabdomyosarcoma	2	16.67
Meningioma atypical	1	8.33
Caring—Length of time		
< 3 months	1	8.33
3–6 months	2	16.67
7–12 months	6	50
> 12 months	3	25
Family caregivers’ Working Status		
Working	5	41.67
Not working	7	58.33
Number of home visit		
< 20 times	5	41.67
> 20 times	7	58.33
Number of telehealth services		
< 20 times	8	66.67
> 20 times	4	33.33

There were three main themes that emerged: [1] the implementation of home-based PPC; [2] the benefits of home-based PPC; and [3] parents’ hopes from the home-based PPC service and their impressions about home-based PPC (Table 4).

**Table 4:** Themes and Categories

Theme	Category
The implementation of home-based PPC	1.Educate families to care for the patient 2.Care delivery 3.Accompaniment at end-of-life phase 4.Bereavement support
The benefits of home-based PPC	1.The management of physical symptoms 2.Motivation and psychosocial support for patients and their families 3.Financial assistance 4.Logistic support
Parents’ hopes from home-based PPC and their impressions about home-based PPC	Parents’ hopes from home-based PPC and their impressions about home-based PPC

**Table 5:** Theme 1. The implementation of home-based PPC

Categories	Sample Quotes
1.Educate families to care for the patient	*“…I learned many new things from the nurses. They taught me how to observe the effect of the morphine, how to write down the administration of morphine on the table (diary)…”* (P6, 36 year-old (hereafter, y.o.) mother of a child with rhabdomyosarcoma) *“… He (the nurse) first demonstrated to me how to do the wound care, then he trained me to do it by myself. He observed what I was doing and gave me feedback until I could do it well. In addition, he told me to administer one or two morphine before doing the wound care so the pain will be less….”* (P10, 53 y.o father of a child with Rhabdomyosarcoma stage IV) *“…Once my son was having diarrhea and vomiting, then I was told and taught about making oral rehydration salt (ORS) so my child won’t suffer from dehydration. Then he (the nurse) told me to stop giving milk temporarily…He also gave me knowledge about medication which I never knew before, such as prontosan, fucidin, to cleanse the infection area…”* (P12, 40 y.o, mother of a child with medulloblastoma)
2.Care delivery	*“…The approach he (the nurse) used when taking care of my son took into account his age and development stage. He let my son explore the medical equipment, before asking for his cooperation as he started to examine him. So, it’s not forcing but respectful of my son…”* (P1, 43 y.o mother of a child with retinoblastoma stage IV and frontoparietal metastases) *“…The nurse communicated well and smoothly, respecting my child’s and parents’ willingness… The nurse will not even go one step further if the family did not allow…”* (P6, 36 y.o mother of a child with rhabdomyosarcoma) *“…The nurse from Rachel House always encouraged us, especially when we were feeling down. They gave us a chance to express our feelings and openly asked us about how they can support us… Nurse R was also acted as a “bridge” to communicate my child’s condition to the doctor at the hospital…”* (P8, 34 y.o mother of a child with neuroblastoma) *“… Nurse D gave us explanation in a way that we could understand, so we could decide about what was needed and important for our child… it was very helpful….”* (P10, 53 y.o father of a child with Rhabdomyosarcoma stage IV)
3.Accompaniment at end-of-life phase	*“…We received explanation before about what might happen to my son. It was hard though, but we appreciated this so much and could prepare ourselves for the worst … When my son had seizure, the explanation became real and the nurse was there to support us…”* (P1, 43 y.o mother of a child with retinoblastoma stage IV and frontoparietal metastases) *“…Nurse A explained to me about the disease pathway and progression. She also informed me about the end-of-life process. When the time came, while I was out of my mind, she was there to remind me that this might be the end-of-life stage that we had discussed. She was there to support me in the difficult time…”* (P6, 36 y.o. mother of a child with rhabdomyosarcoma) *“…The nurse from Rachel House informed me about the signs of end-of-life phase. I thought that was time for us to be prepared for the loss…”* (P7, 39 y.o mother of a child with relapse ALL and central nervous metastases) *“…Even at the end stage of my child’s life, nurse D was there to accompany my family. I was finding many signs, for instance the bluish nail, the breathlessness, and so on. Then I asked nurse D. He answered my questions that these might be symptoms of the end-of-life, then was there to support and comfort me and my husband…”* (P12, 40 y.o mother of a child with medulloblastoma)
4.Bereavement support	*“…The nurse came to the funeral… The nurse even came for the Remembrance Day, a week after my son passed away and it was until the evening…”* (P1, 43 y.o mother of a child with retinoblastoma stage IV and frontoparietal metastases) *“…Nurse A had told me that she will visit us some days after R left us… when she came, my parents were still with us, Nurse A encouraged us to express our feelings and thoughts… when we grieved, Nurse A gave us time and the chance to express our grief, we did not need to avoid or deny it, we could cry if we felt sad, we were allowed to remember our beloved child. She enlightened us that grieving was a normal process…”* (P6, 36 y.o. mother of a child with rhabdomyosarcoma) *“…The nurse visited us about a week after he passed away…The nurse gave me support and encouraged me to heal. I then experienced that Rachel House gave me support even after I lost my child, the nurse always checked up on me…”* (P7, 39 y.o mother of a child with relapse ALL and central nervous metastases) *“…Nurse R always reminded me that it was the Higher plan. My child wanted to rest well, so I did not need to feel guilty even though I was sad about losing him….”* (P9, 40 y.o mother of a child with Meningioma Atypical grade II)

### Theme 1: The implementation of home-based PPC

The first theme that emerged from the participants was the type of care they received through the home-based PPC. There were four categories constructing this theme: how nurses educate parents to provide care for their sick child, how nurses provide care for the patients and families, how nurses accompany patients and parents during the end-of-life phase, and finally, how nurses support parents in their grieving and bereavement process (Table 5).

### Theme 2: The benefits of the home-based PPC

The participants conveyed that the home-based PPC provided several benefits. The benefits were holistic in nature, managing the physical symptoms of the patients, providing emotional and psychosocial support for the patients and their families, and finally, the provision of logistical support and financial assistance.

Three categories emerged in Theme 2. The first category was chosen because all twelve participants expressed that the home-based PPC service helped them to better care for and manage their children’s physical symptoms and issues. These could be seen from several statements in Table 6. The second category emerged because ten participants expressed that the home-based PPC service also provided them with emotional and psychosocial support, both for their child and also the family. The statements from the participants about this appear in Table 6 below. The third category was chosen because there were nine participants who mentioned that the home-based PPC service provided them with financial relief. They experienced economic relief while receiving the home-based PPC service. The fourth category appeared because there were seven participants who conveyed that the home-based PPC service had a positive impact on their lives while caring for their sick child. This is reflected in several statements in Table 6 below. The final category in this second theme was the notion from parents that they also received support in logistics and essential items from the home-based PPC service. Eleven participants shared their experience in receiving these logistics support. These are illustrated in the statements in Table 6 below.

**Table 6:** Theme 2. The benefits of home-based PPC

Categories	Sample Quotes
1.The management of physical symptoms	“…the pain had been controlled for the last one month. It was managed well and I think it was because of Nurse A and her team’s help… her urine output was getting lower and I worried that it was a sign of urine retention. So, I discussed with Nurse A about the possibility of a consultation with the doctor…” (P6, 36 y.o mother of a child with rhabdomyosarcoma) “…she also helps us continuously. When she visited us, she did the wound care…” (P7, 39 y.o mother of a child with relapse ALL and central nervous metastases) “…it was when nurse R visited us… I saw my child, N, was bleeding profusely and vomiting. I was shocked, trembling, and scared. Thankfully she was there and helped us with this horrible condition…” (P8, 34 y.o mother of a child with neuroblastoma)
2.Motivation and psychosocial support for patients and their families	“…I remember, I chatted via phone calls or WhatsApp texting with the nurse at night, and it’s soothing. I think it is very important for the family with a sick child, especially a long-term sickness. This kind of support is very helpful…” (P2, 50 y.o mother of a child with neuroblastoma) “…but N (my child) was getting brighter after knowing Rachel House’s team. We did not expect this. We salute the team for being able to lift N’s spirit because nurse D was very likeable… N was very happy with Nurse D. He survived quite long, about 1 year after being diagnosed… I think one of the most important factors is trust and I trust that Rachel House could help me.” (P1, 43 y.o mother of a child with retinoblastoma stage IV and frontoparietal metastases) “…The sharing session was very helpful for me. I could share not only about my child’s condition and treatment but also about emotional things. I felt at peace because I knew that other than my family, someone was there to soothe and strengthen me. So, the team was calming for us, especially at the terminal phase when we were not ready for the uncertainties to come…” (P12, 40 y.o mother of a child with medulloblastoma)
3.Financial assistance	“…after receiving the home-based PPC, I hoped to continue to care for my child at home… then I could work from home to help my husband earn money for our family needs…” (P3, 43 y.o. mother of a child with relapse ALL) “…I could say we received economic benefit… because it was true that our expenses for medical care reduced after we received the home-based PPC…I also did not have to worry about the hospital bills if he was admitted in the hospital…” (P6, 36 y.o. mother of a child with rhabdomyosarcoma) “…Rachel House was helping us a lot by giving us that expensive morphine. Moreover, they also gave us other medications that was needed which, when combined, cost a lot…” (P7, 39 y.o. mother of a child with relapse ALL and central nervous metastases)
4.Logistic support	“…N (my child) also received diapers… When he could not walk and needed a stroller, Rachel House team brought us a stroller. It helped, especially when we needed to go to the hospital …” (P1, 43 y.o mother of a child with retinoblastoma stage IV and frontoparietal metastases) “…they also gave us groceries, and milk. We knew that the donor gave us through Rachel House …” (P3, 43 y.o mother of a child with relapse ALL) “…we received assistance several times, for instance during Ramadan and the pandemic, they sent us groceries…” (P7, 39 y.o mother of a child with relapse ALL and central nervous metastases)

### Theme 3: Parents’ hopes from home-based PPC and their impressions about home-based PPC

Parents hoped that the home-based PPC would help improve the quality of life of their children with the assistance of competent professionals who provided quality care through the service. Therefore, parents expressed their feelings and thoughts during and after receiving the home-based PPC experience. All parents experienced an unforgettable time and expressed their gratitude as can be seen in the statements in Table 7.

**Table 7:** Theme 3. Parents’ hopes from home-based PPC and their impressions about home-based PPC

Categories	Sample Quotes
Parents’ hopes from home-based PPC and their impressions about home-based PPC	“…Rachel House team was memorable, the home-based care they provided exceeded our expectation…We received home-based PPC for one year, it was unforgettable and amazing…” (P1, 43 y.o mother of a child with retinoblastoma stage IV and frontoparietal metastases) “…Nurse E always gave me support and suggestions. She hugged me to strengthen and encourage me. Rachel House was so helpful. If I asked something, they will always give me answers and solutions…” (P5, 34 y.o. mother of a child with ALL) “…I could only say that this was not an ordinary home-care, it’s extraordinary. I understood about palliative care because they told us, they engaged us in 4s, they respected us, listened to us. I thought this was one of the most needed skills from health care providers, to be willing to really listen to us, asking about our thoughts and feelings, validating and offering help. That’s the reason I thought Rachel House was extraordinary. Even after the first meeting, I could conclude that this palliative care was more than okay…” (P6, 36 y.o. mother of a child with rhabdomyosarcoma)

## Discussion

This study highlights that the home-based PPC delivered by Rachel House provides holistic care for the patients, as well as for their parents. Parents received education to help them provide care for their sick child and was supported during the end-of-life phase, continuing through to their grieving and bereavement process. Even so, it is not easy for parents to initially accept the home-based PPC service [12]. Acceptance of PC in Indonesia remains a challenge due to cultural issues [27] and also family-related expectations such as: a) caring for a sick family member is a family obligation, b) family believes that they must continue to seek a cure and accepting palliative care equals giving up, c) pressure from extended families and feelings of guilt and regrets, and d) family is concerned that accepting PC means they have to forego treatment. Additionally, there is a prevalent belief that parents should surrender to the will of God and not attempt any interventions [28]. However, home-based PPC is gaining acceptance from families with children living with life-threatening illness as they choose home as the preferred care site.

The implementation of home-based PPC could help parents in dealing with many problems in caring for their children living with life-threatening illnesses. Once the family agrees to receive the home-based PPC service, a nurse from Rachel House is allocated to the patient and visits the family regularly. Some of the greatest fears that parents have while caring for their children living with life-limiting or life-threatening illness at home is not knowing what to do, nor how to cope with the children’s daily needs and deal with their deteriorating condition [29–31]. The central tenet of holistic care for both the patient and family in PPC is implemented even at the home setting [29–31]. The goals of PPC are to reduce the suffering and improve the quality of life of children living with chronic and terminal illness [30, 32, 33] and provide support for their parents and other family members [30, 34].

In this study, we discovered that parents of children living with cancer experienced several benefits from receiving the home-based PPC service, including parents received help to care and manage the physical issues that occurred as their children’s condition deteriorated and particularly, at the end-of-life phase. For example, one mother expressed her feelings of relief because the Rachel House’s nurse was by her side to hold her hand as her child was at the end of his life. She felt supported and did not feel alone during these difficult times. Furthermore, parents experienced positive impacts on their own wellbeing through the home-based PPC service, such as, having peace of mind, reduced anxiety, time effectiveness, and saving energy. The care provided by the nurses also included support for the patients’ basic and essential needs and even financial assistance. These benefits which had been experienced by the families showed that the home-based PPC service would be beneficial not only for the sick children but also their family. This finding is similar to the previous studies [29–33, 35], and also supported by Kringos et al.’s [18] study that mentioned home-based PPC is essential for a better healthcare quality management, public accountability and patient preferences.

In addition, parents mentioned that they were comforted knowing whom to contact and where to seek help if something were to happen with the children. Parents were also assured by the nurses who would regularly follow-up on the children’s and family’s conditions. Moreover, parents could openly share their feelings, thoughts and fears with the nurses. This assistance might also be comforting for the parents knowing that they were not alone, and there will be someone capable to help them deal with uncertainties [30]. This might also manifest as motivational or psychosocial support for patients and their families.

In this study, parents expressed their hopes from the home-based PPC service and their impressions about home-based PPC. Parents mentioned that their interactions with the Rachel House’s nurses played an important role in meeting the care needs of their sick child. The nurses’ competence and their presence seemed to help the parents feel confident and assured to be at home [12, 30, 31]. Parents could discuss important matters with the nurses because they were knowledgeable and skilled. The professional caregivers soothe the patients’ and their parents’ anxieties and reduce their burden by showing their competence and compassion [36–38]. Parents named several attributes that were noticeable in the nurses, for instance, good clinical skills in providing care, excellent communication skills throughout the care process (both to patients and the family); good knowledge about the children’s condition and disease trajectory, and their effectiveness in helping bridge the communication between patients and their pediatric oncologists.

Parents as family caregivers in this study mentioned that the home-based PPC helped build their capacity as caregivers. As mentioned in another study [31], home-based PPC nurses were not only helping the patients with symptom management, but they also helped build the capacity of the parents to provide daily care for their sick child. The parents conveyed that they were informed about what their children needed on a daily basis and even trained to apply several basic skills to care for the child, such as feeding by nasogastric tube, wound care, pain management, hyperthermia management, and bleeding management. This gave the parents the opportunity to help their sick child as best as they could and enhance parent–child bonding [31]. The provision of PPC could also be associated with improving the psychosocial outcomes in the family [30, 39]. This positive impact is possible because during the delivery of palliative care, parents were trained and equipped with the skills to manage the child’s symptoms. Moreover, rather that receiving intensive treatment at the end-of-life that may increase the suffering of the child and reduce the time with their parents, the holistic PPC is focused on managing the child’s pain and symptoms while preparing the parents for the end-of-life, which allows parents to spend these precious remaining days with their children [30, 39].

### Strengths and Limitations of the Study

This is the first study that explores the experiences of family caregivers in receiving home-based PPC in Indonesia. Given the paucity of PPC in Indonesia, the findings of this study could provide insights into the beneficiaries’ experiences and their needs, rather than that of the medical professionals’, particularly from caregivers of children diagnosed with cancer.

The first limitation of this study is that most of the participants were women (mothers). This is because in Indonesia, women are the dominant family caregivers, whether it be caring for children or elderly, and whether they are healthy or sick. Based on this women-dominant factor in the participants, this study cannot be generalized considering that the experiences of mothers caring for sick children are not necessarily the same as the experiences of fathers caring for sick children. The second limitation of this study is that all the children were diagnosed with cancer, and therefore this study cannot be generalized considering that the experiences of family caregivers caring for sick children with cancer might be different from the experiences of those caring for children with other conditions such as neurologic, pulmonary, genetic. Another limitation is that while caring for terminally-ill children must be unforgettable and deeply remembered by the parents, the recall bias could be possible with the passage of time since the moment of loss.

## Conclusions

Family caregivers acknowledged the importance of the home-based PPC service and experienced several benefits in the physical, psychosocial, emotional, spiritual and financial aspects. The sustainability and the expansion of the Rachel House’s home-based PPC service can help increase its contribution towards improving the quality of life of children living with life-threatening illness and their families, particularly in accompanying families facing the difficult times of losing their loved ones.

## Data Availability

The datasets used and analyzed during the current study are not publicly available due to restraints of the ethical permit. Some data may be available from the corresponding author upon reasonable request.

## Abbreviations

AAP: American Academy of Pediatrics
ALL: Acute lymphocytic leukemia
COREQ: Consolidated Criteria for Reporting Qualitative Research
HBPC: Home-based Palliative Care
HCPs: Health Care Professionals
NHPCO: National Hospice and Palliative Care Organization
PPC: Pediatric Palliative Care
WHO: World Health Organization
